# Analysis of cataract-regulated genes using chemical DNA damage induction in a rat *ex vivo* model

**DOI:** 10.1371/journal.pone.0273456

**Published:** 2022-12-07

**Authors:** Risa Yamaoka, Fumito Kanada, Masaya Nagaya, Masaru Takashima, Yoshihiro Takamura, Masaru Inatani, Masaya Oki

**Affiliations:** 1 Department of Industrial Creation Engineering, Graduate School of Engineering, University of Fukui, Fukui, Japan; 2 Department of Ophthalmology, Faculty of Medical Sciences, University of Fukui, Fukui, Japan; 3 Life Science Innovation Center, University of Fukui, Fukui, Japan; University of Colorado Denver School of Medicine, UNITED STATES

## Abstract

Although cataracts affect almost all people at advanced age and carry a risk of blindness, the mechanisms of cataract development remain incompletely understood. Oxidative stress, which is a causative factor in cataract, results in DNA breakage, which suggests that DNA damage could contribute to the formation of cataracts. We developed an *ex vivo* experimental system to study changes in gene expression during the formation of opacities in the lens by culturing explanted rat lenses with Methylmethanesulfonate (MMS) or Bleomycin, which induce DNA damage. Lenses cultured using this experimental system developed cortical opacity, which increased in a concentration- and time-dependent manner. In addition, we compared expression profiles at the whole gene level using microarray analysis of lenses subjected to MMS or Bleomycin stress. Microarray findings in MMS-induced opacity were validated and gene expression was measured from Days 1–4 using RT-qPCR. Altered genes were classified into four groups based on the days of peak gene expression: Group 1, in which expression peaked on Day 1; Group 2, in which expression peaked on Day 2; Group 3, in which expression progressively increased from Days 1–4 or were upregulated on Day 1 and sustained through Day 4; and Group 4, in which expression level oscillated from Days 1–4. Genes involved in lipid metabolism were restricted to Group 1. DNA repair- and cell cycle-related genes were restricted to Groups 1 and 2. Genes associated with oxidative stress and drug efflux were restricted to Group 2. These findings suggest that in temporal changes of MMS-induced opacity formation, the activated pathways could occur in the following order: lipid metabolism, DNA repair and cell cycle, and oxidative stress and drug efflux.

## Introduction

Cataract is a disease in which the lens becomes opaque and vision is impaired. Despite the widespread use of phacoemulsification-aspiration in developed countries, cataracts are still the main cause of acquired blindness, mainly in developing countries [1]. The detailed molecular mechanisms of disease have not been elucidated. Pharmacologic interventions have yet to be developed [2], and the only clinically available treatment is surgical removal of the lens and replacement with an artificial lens.

UV radiation [3, 4], diabetes [5], and oxidative stress [6, 7] are known to contribute to cataract pathogenesis, and various animal models of cataracts have been used to elucidate the mechanisms and conduct drug screening.

Microarray analysis of lens epithelial cells from mice revealed that Orthodenticle homeobox 2 (*Otx2*) expression was increased after UV-irradiation [8]. In addition, immunostaining studies of lenses after UV-irradiation revealed increased expression of active caspase 3 [9]. In an *in vivo* experimental system using explanted rat lenses, administration of caffeine eye drops inhibited UV irradiation-induced formation of lens opacity [10]. Furthermore, UV-induced cataract formation was suppressed in Vitamin E-fed rats [11]. UV radiation exposure of rat lenses cultured *ex vivo* results in dose-dependent increases in opacity [9]. In *ex vivo* UV experiments, ROS-induced damage occurs in the lens, and UV-induced opacity is prevented by pyruvate [12] or caffeine [13].

Two animal models are used to study effect of diabetes on cataracts, including chemical induction of type 1 diabetes with streptozotocin in rats, and simulating the effects of hyperglycemia by supplementing a galactose-rich diet in rats. In an *ex vivo* experimental system, rat lenses are cultured *ex vivo* in galactose-enriched media to induce cataractogenesis, which could be related in part to abnormal ascorbic metabolism in the lens [14]. In addition, galactose-induced opacity is suppressed by histone acetyltransferase (HAT) inhibition and worsened by histone deacetylase (HDAC) inhibition, suggesting the involvement of epigenetic modifications [15].

In an *ex vivo* experimental system using selenite, chrysin, a type of polyphenol, alleviates opacity formation [16]. An *in vivo* experimental system using subcutaneous injection of sodium selenite also identified a protective effect of the polyphenol compound rosmarinic acid [17]. Thus, important causative factors and potential therapeutic targets have been identified in each experimental system, but drugs with antioxidant capacity tend to be effective in a wide range of experimental systems.

Oxidative stress is considered to be an important factor in the development of cataracts by all causes in humans [18], and is caused by reactive oxygen species (ROS) generated by cellular energy metabolism and by acute or prolonged exposure to UV light or ionizing radiation [19]. To directly investigate the effects of oxidative stress on cataracts, an *ex vivo* experimental system in which lenses are removed from rats and subjected to H_2_O_2_-induced oxidative stress is utilized. In this experimental system, treatment with AL-3823A, an antioxidant mimetic of glutathione peroxidase, prevents opacity formation [20]. On the other hand, a study evaluating the effects of H_2_O_2_ on lens epithelial cells identified a dramatic decrease in cell viability, increased aging-related factors, and pathological changes in cell morphology [21].

Oxidative stress damages cellular macular molecules through mechanisms such as protein modification, lipid peroxidation, and DNA base damage and fragmentation [18]. Although oxidative stress can cause both single- and double-strand DNA breaks [18, 22, 23], few studies have evaluated the direct effects of lens cell DNA damage on cataract formation [18, 24–29]. Ataxia telangiectasia mutated (ATM) and Ataxia telangiectasia and Rad3-related (ATR) are protein kinases that play important roles in the DNA damage response, and activate the transcription factor p53 as their primary target [30]. p53 activates transcription of downstream factors such as p21, leading to cell cycle arrest and, if strongly activated, apoptosis [31]. In our previous study, we reported that galactose-induced cataract formation is inhibited by Atm inhibitors, and that Atm is involved in cataract formation [15]. DNA damage is also increased in lens epithelial cells of cataract patients [25]. UV irradiation of lens epithelial cells, which causes cataracts, induces DNA damage and results in activation of ATM, which is associated with increased p21 and cell cycle arrest [32]. Collectively, these findings suggest that DNA damage could be related to cataract formation.

Methylmethanesulfonate (MMS), a DNA damage-inducing agent used in the present study, causes single-strand breaks by alkylating DNA, predominantly at adenine and guanine, resulting in base mispairing and replication blocking [33]. Bleomycin causes DNA damage by binding to metal ions such as iron to form a complex that produces ROS, which cause single- and double-strand DNA breaks between the 3’-4’ bonds of deoxyribose [34].

In the present study, we developed a new experimental system to induce opacity in rat lenses cultured *ex vivo* by adding MMS or Bleomycin to culture medium. This system has two advantages; one, it rapidly induces opacity in rat lens, and two, compared to other ex vivo experimental systems, it excludes off-target effects and allows a more DNA damage-focused mechanistic analysis. This newly developed experimental system of cataractogenesis will make a broad contribution to elucidating the role of DNA damage in the pathogenesis of cataracts.

## Materials and methods

### Lens enucleation and culture

We used 6-week-old male Sprague Dawley rats. Rats were euthanized with CO_2_ asphyxiation, and eyeballs were removed. Next, we enucleated lenses in PBS with using a dissection microscope. Enucleated lens were cultured in 2 mL serum-free M199 medium (Sigma) containing 0.1% BSA [35]. MMS (Nakalai tesque) or Bleomycin (Tokyo chemical industry) were added to the culture medium at final concentrations of 1 mM and 800 μg/mL, respectively. Culture medium was changed once daily, and fresh drug was added to the medium every time it was changed. All lenses were maintained at 37°C in a humidified incubator with 95% room air and 5% CO_2_. On the last day of culture, we photographed lenses with a microscope using a DP58 camera (Olympus) attached to an SZX12 stereomicroscope in a 35 mm dish containing 7 mL PBS. All experiments were approved by the Animal Research Committee of the University of Fukui (Approval number: 28091) and were conducted in accordance with the University of Fukui Animal Care and Use Regulations and Association for Research in Vision and Ophthalmology Statement for the Use of Animals in Ophthalmic and Vision Research. The findings of the study were reported in accordance with ARRIVE guidelines.

### RNA extraction

After *ex vivo* assays, lenses were homogenized in TRIzol reagent (Thermo Fisher Scientific), and RNA was extracted according to the TRIzol manufacturer’s protocol. RNA was eluted in in DEPC water in and stored at -20°C until analysis.

### Reverse transcription and RT-qPCR

Lens RNA was reverse transcribed to cDNA with a reverse transcription kit (Applied Biosystems). After reverse transcription, the amount of cDNA was measured by RT-qPCR with SYBR Green master mix (Applied Biosystems), and threshold cycle data were normalized using *Gapdh*. The primer sequences are shown in S1 Table.

### Microarray data analysis

Microarray analysis of five lens sample groups were conducted: A control lens cultured with normal medium for 4 days, lenses treated with 1 mM MMS for 2 and 4 days, and lenses treated with Bleomycin for 2 and 6 days (n = 1/group). Microarray analysis was performed as previously described [15]. A rat Gene Chip Gene 2.0 ST Array (Thermo Fisher Scientific) was used for microarray analysis. Data were analyzed using R. The RMA (Robust Multi-array Average) algorithm was used to normalize data, and unnamed genes and genes with low signal values (Max signal < 5.00 for all samples) were excluded. Heat maps and PCA plots were generated for included genes. In addition, genes that were upregulated greater than 2-fold relative to control were extracted. Expression levels of these genes were quantitatively measured using RT-qPCR (n = 3). Gene networks were generated using STRING (https://string-db.org/), a database of protein–protein interactions. Data are available at GEO (Gene expression omnibus) under the accession number GSE194317 (https://www.ncbi.nlm.nih.gov/geo/query/acc.cgi?acc=GSE194317).

## Results

### MMS and Bleomycin-induced lens opacity

**Fig 1:**
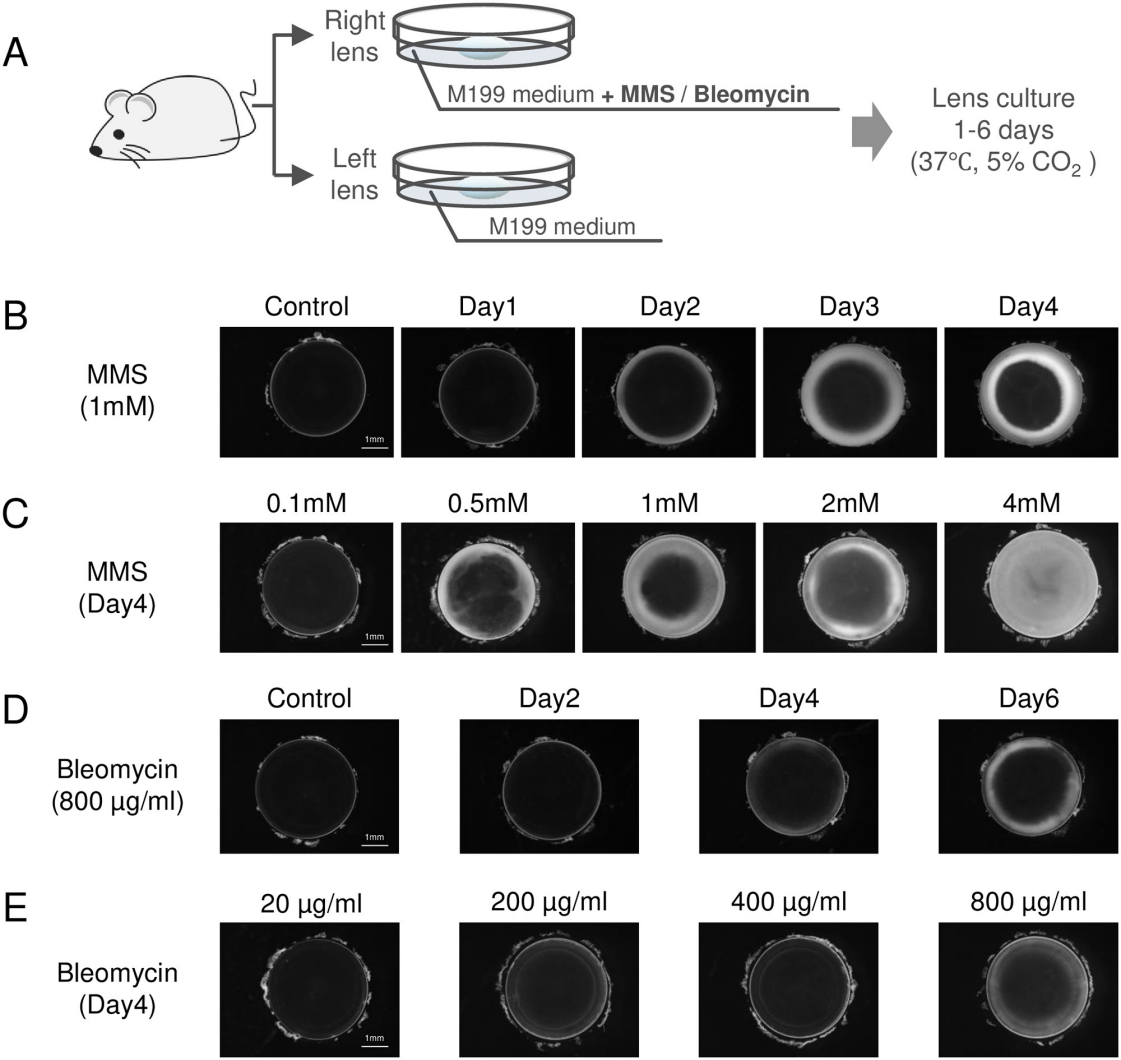
MMS and Bleomycin induce rat lens opacity in time- and concentration-dependent manners. **(A)** Schematic of the *ex vivo* experimental system used to induce opacity in rat lenses with MMS or Bleomycin treatment. Lenses were enucleated from 6-week-old male Sprague Dawley rats and cultured for 1–6 days. From each individual animal, one lens was cultured in medium containing MMS or Bleomycin, and the lens of the contralateral eye was cultured in vehicle control medium. **(B)** Opacity induced by incubation of lenses in medium containing MMS (1 mM) for 1–4 days. Control lenses were cultured in medium without MMS for 4 days. **(C)** Opacity induced by culture of lenses in medium containing 0.1–4 mM MMS for 4 days. **(D)** Opacity induced by incubation of lenses in medium containing Bleomycin (800 μg/mL) for 2–6 days. Control lenses were cultured in medium without Bleomycin for 4 days. **(E)** Opacity induced by culture of lenses in medium containing 20–800 μg/mL Bleomycin for 4 days.

In our prior study, we reported that inhibitors of ATM, a key factor in the DNA damage response, prevent cataractogenesis [15]. Therefore, we hypothesized that the DNA damage response could contribute to the development of cataracts, and sought to determine if subjecting lenses to the DNA damage inducers MMS and Bleomycin would induce lens opacity. Lenses were enucleated from 6-week-old Sprague Dawley rats and cultured in medium containing MMS or Bleomycin for 1–6 days (Fig 1A). MMS induced opacity in the cortex of the lens, which was time- (Fig 1B) and concentration-dependent (Fig 1C), and progressed from the equatorial region to the center of the lens. Bleomycin also induced opacity in a time- and concentration-dependent manner (Fig 1D and 1E). Bleomycin-induced opacity was very thin at low concentrations and short incubations, manifesting as a thin and narrow white ring in the equatorial region (Fig 1D and 1E). Bleomycin-induced opacity progressed with increased incubation time and drug concentrations and spread from the equatorial toward the center of the lens.

**Fig 2:**
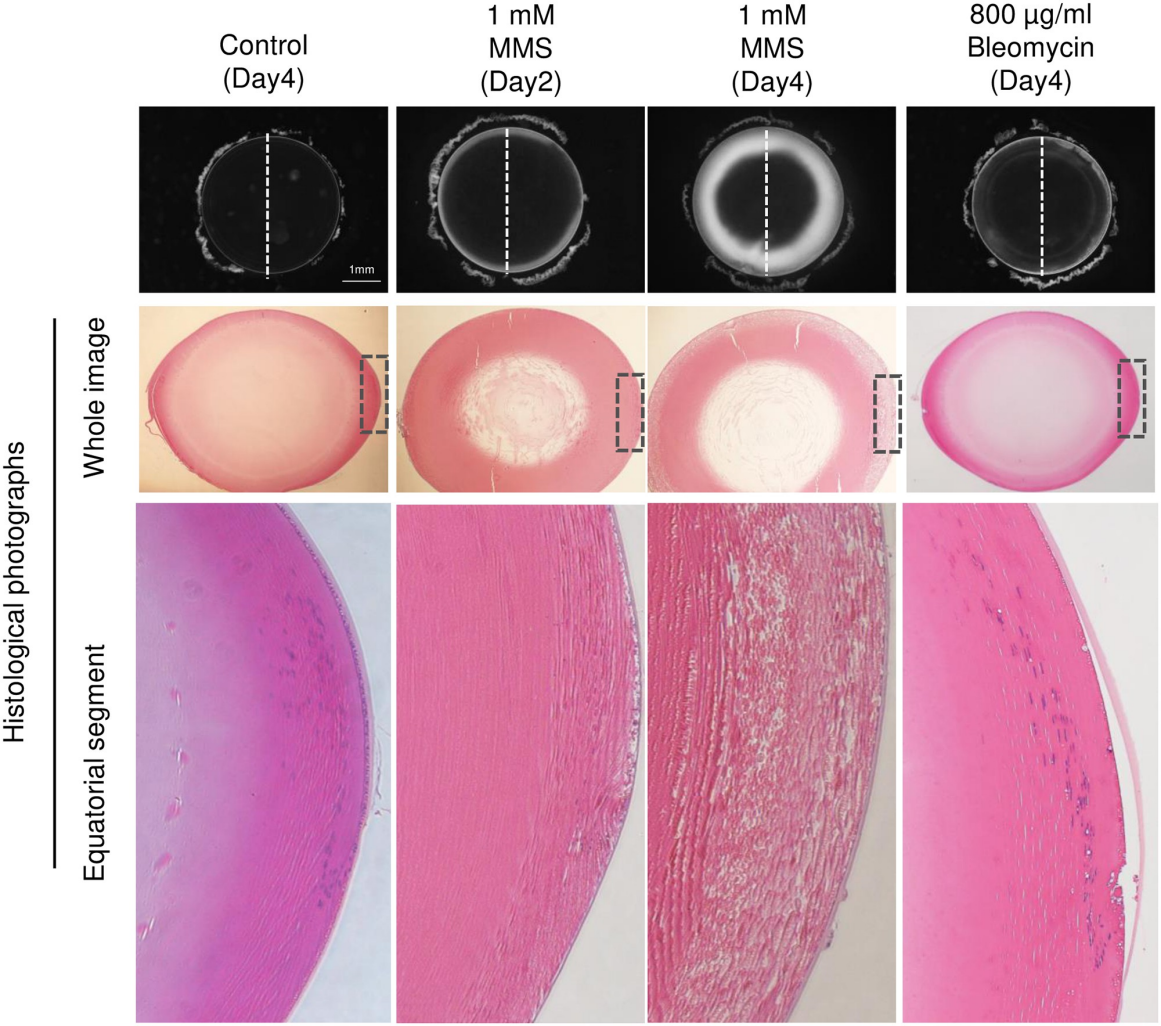
Cross-sectional morphology of lenses subjected to MMS or Bleomycin. The images in the top row show micrographs of the lenses. The images in the middle row show whole images of hematoxylin and eosin (H&E) stained paraffin cross-sections that were sectioned in the direction indicated by the dashed line on the micrographs (HE). The images in the bottom row are enlarged images of the equatorial part of the section image in the middle row, denoted by areas surrounded with dashed lines.

We prepared paraffin sections of control lens cultured in medium without MMS or Bleomycin, lenses treated with MMS, and lenses treated with Bleomycin, and stained sections with hematoxylin and eosin for morphological analyses (Fig 2). Lenses treated with MMS exhibited an overall decrease in cell density in the superficial layer of the lens. Comparing lenses cultured at the same MMS concentration (1 mM) for 2 and 4 days, the surface layer cell density decreased as incubation time increased. In MMS-treated lenses, opacity increased as lens surface cell density decreased, suggesting that MMS-induced opacity was due to decreased cell density in the superficial layer of the cortical area. In the cross-section images of lenses treated with Bleomycin for 4 days, gaps were present between cells in the superficial layer of the cortical region of the lens, and cell density was reduced. These gaps were present not only in the transitional zone but also in the entire superficial layer. Observation of the lens interior using paraffin sections revealed that MMS- and Bleomycin-induced opacities were similar, and were likely caused by drug-induced decreases in cell density in the superficial layer of the cortex.

### Interrelationships between gene expression profiles of lenses with MMS or Bleomycin-induced opacity

**Fig 3:**
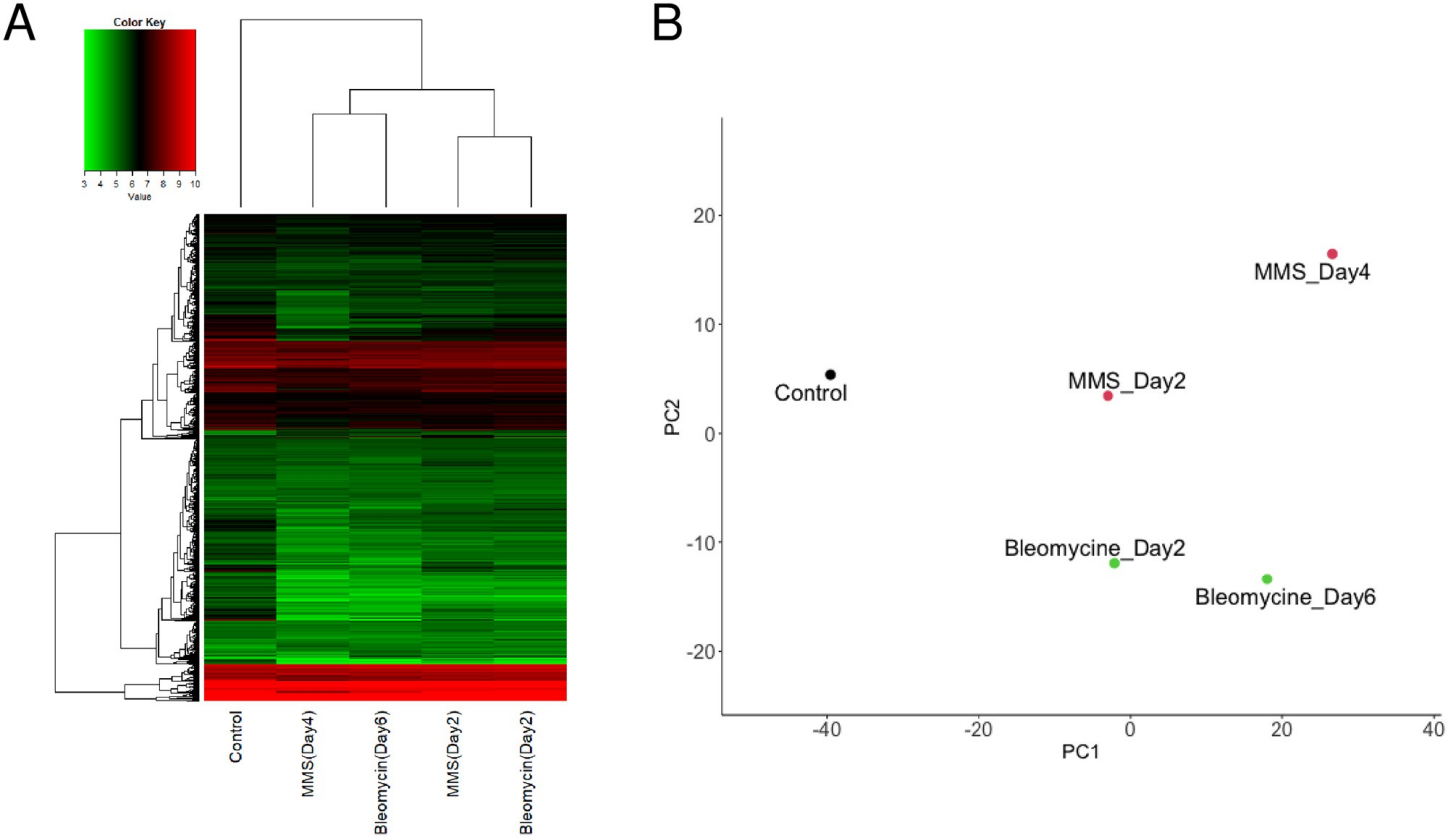
Interrelationships between expression profiles of lenses with MMS or Bleomycin-induced opacity. **(A)** Heatmap showing the relationship between gene expression profiles of Control, MMS Day 2, MMS Day 4, Bleomycin Day 2, and Bleomycin Day 6 groups. In the vertical direction, 4,649 genes remained after outliers (probes without a corresponding gene name and probes with signal values < 5) were excluded. Genes with decreased signal values are shown in green, and genes with increased signal values are shown in red. The clustering at the top indicates the similarity between samples, and the lower the clustering hierarchy, the higher the similarity between clusters. **(B)** A PCA plot showing the relationship between gene expression profiles of Control, MMS Day 2, MMS Day 4, Bleomycin Day 2, and Bleomycin Day 6 groups. The signal values of 4,649 genes, the same as in (A), were used to generate the PCA map. A closer distance between the sample dots indicates a higher degree of similarity between gene expression profiles.

To investigate changes in gene expression levels when lens opacity was induced by MMS or Bleomycin, microarray analysis was performed using mRNA from control, MMS Day 2, MMS Day 4, Bleomycin Day 2, and Bleomycin Day 6 lenses. First, we compared overall expression profiles using heat maps and PCA plots (Fig 3). In the heat map, the pair of MMS (Day 4) and Bleomycin (Day 6) groups and the pair of MMS (Day 2) and Bleomycin (Day 2) groups were clustered close to one another (Fig 3A). The control was clustered farthest away from the other treatment groups. In the PCA plot, the samples were plotted at increasing distances away from the control group. The distances increased progressively in ascending order as follows: MMS Day 2, Bleomycin Day 2, MMS Day 4, and Bleomycin Day 6 (Fig 3B). The difference in incubation time affected the expression profiles more strongly than did the difference in drug treatments between MMS and Bleomycin. These findings suggest that MMS and Bleomycin have similar effects on changes in the overall lens gene expression profiles. The above results prompted subsequent detailed analysis of lenses treated with MMS.

### Identification of genes significantly upregulated by MMS

**Fig 4:**
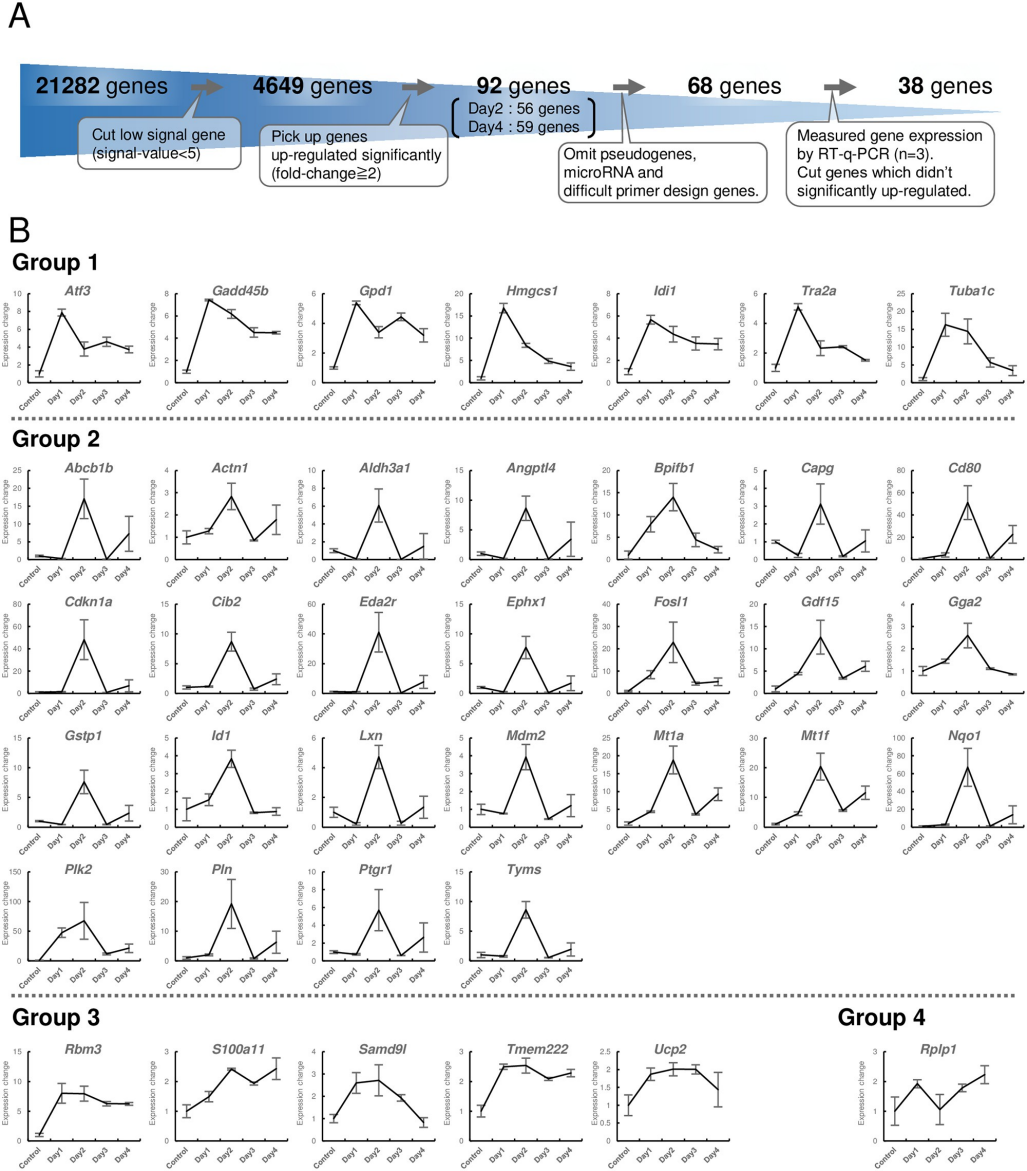
Quantitative analysis of changes in gene expression levels. **(A)** The process of narrowing down genes that were significantly altered by MMS treatment. **(B)** Quantitative gene expression analysis by RT-qPCR (biological replicates, n = 3 /group) (S1 Fig). Gene expression levels of 38 genes extracted by the method in (A) were measured at 1, 2, 3, 4 days after MMS treatment and in Control samples, Changes in gene expression levels over time were tracked and are represented in a line graph. Genes were classified into four groups according to the pattern of expression level changes over time.

To investigate the genes involved in the formation of MMS-induced opacity, genes with significantly changed expression levels were extracted based on the microarray results of MMS Day 2 and MMS Day 4 samples. First, after deletion of probes without gene names among 36,685 probes, we excluded 21,282 genes with low signal values, narrowing down the list to 4,649 genes. Then, 56 genes with more than 2-fold upregulation were extracted in comparison with Control and MMS Day 2, and 59 genes with greater than 2-fold increase were extracted in comparison with control and MMS Day 4. Accordingly, 92 total genes were categorized as being upregulated by MMS treatment, as some genes were extracted from both Day 2 and Day 4 (Fig 4A, S2 Table). Next, the expression levels of these genes were quantitatively measured by RT-qPCR. We analyzed 68 genes after eliminating 24 genes that were either pseudogenes or for which primer design was difficult. Thirty-eight genes were found to increase more than 2-fold compared with control, so were in accordance with microarray findings (Table 1, Fig 4A). In addition, to examine changes in the expression levels of these 38 genes over time, RNA was extracted from lenses treated with MMS for 1, 2, 3, and 4 days and gene expression levels were measured (Fig 4B).

**Table 1:** List of genes with significantly altered expression in RT-qPCR (biological replicates, n = 3/treatment group).

Group1	Group2		Group3	Group4
*Atf3*	*Abcb1b*	*Gga2*	*Rbm3*	*Rplp1*
*Gadd45b*	*Actn1*	*Gstp1*	*S100a11*	
*Gpd1*	*Aldh3a1*	*Id1*	*Samd9l*	
*Hmgcs1*	*Angptl4*	*Lxn*	*Tmem222*	
*Idi1*	*Bpifb1*	*Mdm2*	*Ucp2*	
*Tra2a*	*Capg*	*Mt1a*		
*Tuba1c*	*Cd80*	*Mt1f*		
	*Cdkn1a*	*Nqo1*		
	*Cib2*	*Plk2*		
	*Eda2r*	*Pln*		
	*Ephx1*	*Ptgr1*		
	*Fosl1*	*Tyms*		
	*Gdf15*			

Lists of genes grouped according to their expression patterns over time. Group 1 comprises genes that showed a trend of increased expression on day 1 and then a decrease in expression. Group 2 comprises genes that showed a trend of increased expression peaking on day 2 and then a decrease in expression on days 3 and 4. Group 3 comprises genes whose expression tended to increase gradually from day 1 to day 4, or whose an increase in expression was maintained from day 1 to day 4. Group 4 comprises a group of genes whose expression levels repeatedly increased and decreased from day 1 to day 4.

When expression level changes were plotted on a line graph, they were categorized into four patterns. Group 1 showed a trend of increased expression on Day 1 that subsequently decreased, and seven genes were categorized in this group. Group 2 showed a trend of increased expression peaking on Day 2 with subsequent decreasing expression on Days 3 and 4, and 25 genes were categorized in this group. Group 3 showed progressively increasing expression from Days 1 to 4, or maintained upregulated expression levels from Days 1 to 4, and five genes were classified in this group. Group 4 showed oscillating up- and downregulation from Days 1 to 4, and one gene was classified in this group. During the initial period (Day 1), lens opacity did not change appreciably, but the expression of some genes changed significantly.

### Functional analysis of genes significantly increased by MMS

**Fig 5:**
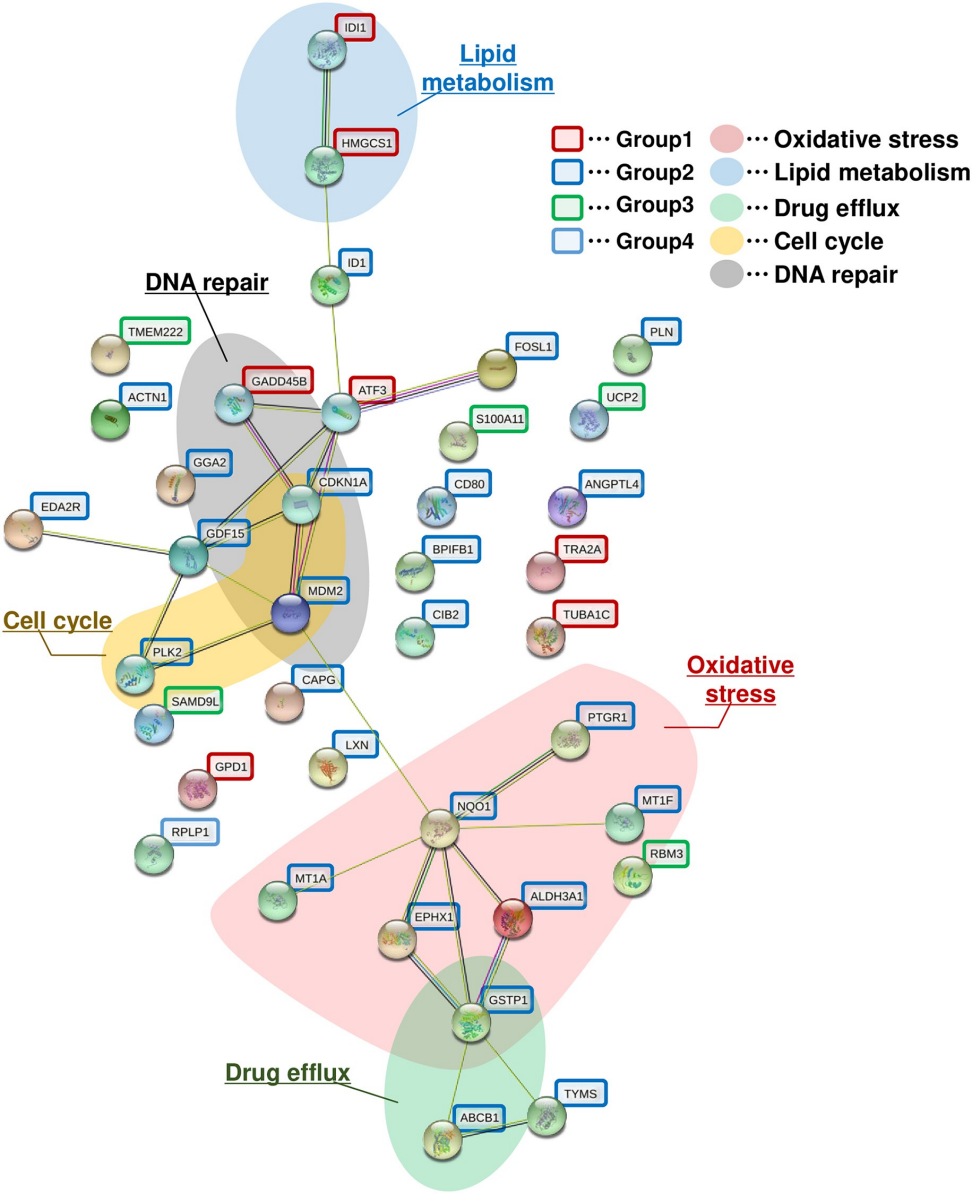
Functional protein association networks established by STRING. Functional protein association networks of 38 genes quantified over time during Days 1–4 of MMS treatment were generated using STRING analysis. Genes belonging to Groups 1, 2, 3, and 4 are circled in red, blue, green, and yellow, respectively. Areas where multiple genes related to oxidative stress, lipid metabolism, drug efflux, cell cycle, and DNA repair were clustered together and were filled with red, blue, green, yellow, and gray, respectively.

To determine which pathways were activated by MMS-induced DNA damage to provoke lens opacity, based on the results of changes in gene expression levels over time obtained by RT-qPCR, STRING analysis was performed on the 38 genes that were significantly increased in RT-qPCR findings. Fig 5 shows the functional protein association networks based on interactions predicted by the known biological functions of the genes and proteins. Twenty of 38 genes were linked to other genes in the network. Based on a literature search of the known biological functions of these genes, we determined that genes related to the oxidative stress response (*Aldh3A1*, *Ephx1 Gstp1*, *Mt1a*, *Mt1f*, *Nqo1*), cell cycle (*Cdkn1a*, *Mdm2*, *Plk2*), DNA damage (*Cdkn1a*, *Gadd45b*, *Mdm2*), drug efflux (*Abcb1b*, *Gstp1*), and lipid metabolism (*Hmgcs1*, *Idi1*) were clustered together in close proximity as more relevant genes. Furthermore, the gene groups related to DNA repair and the cell cycle were distributed in close proximity to one other, and the gene groups related to oxidative stress and drug efflux were also distributed in close proximity to one other. Genes related to lipid metabolism were placed apart from other functional groups. This suggested a link between DNA repair and the cell cycle, and a link between the oxidative stress response and drug efflux in the process of MMS-induced lens opacity formation. In addition, the group of genes involved in lipid metabolism included only genes belonging to Group 1, which exhibited peak upregulation on Day 1. The group of genes related to DNA repair and the cell cycle was a mixture of genes from Group 1 and genes from Group 2, which exhibited peak upregulation on Day 2. Genes associated with oxidative stress and drug efflux included only Group 2 genes. This suggests that in temporal changes of MMS-induced lens opacity formation, the pathways may be induced in the following order: lipid metabolism, DNA repair and the cell cycle, and oxidative stress and drug efflux.

## Discussion

As model systems for cataract research, lens opacity can be induced by culturing explanted rat lens under various conditions, including galactose-containing medium [14, 15], H_2_O_2_ [20], selenite [16], and UV light exposure [12, 13]. However, in the present study, we established an experimental system to induce opacity in rat lenses by inducing DNA damage with MMS or Bleomycin treatment. External factors such as UV light exposure are known to cause cataracts, which is thought to result from physical DNA damage that triggers apoptosis of lens epithelial cells (LECs) [36, 37]. Prior reports have used animal models of UV light exposure [8, 9, 38] and *ex vivo* models of UV light exposure in cultured lenses [12, 13], but the use of UV light makes it difficult to control changes over time and intensity, and thus is not ideal as a drug screening model. However, the system developed in the present study utilizes drugs that cause DNA damage, allowing analysis of time- and concentration-dependent effects. Since the speed of cataract progression can be easily controlled in our system, it is possible to screen for drugs that inhibit cataract progression or drugs that reverse cataracts once they have formed.

Lenses treated ex vivo with MMS or Bleomycin developed an annular white opacity in the cortical equatorial region. In a previous study, in lenses cultured in medium containing galactose and H_2_O_2_, and in an ex vivo experimental system in which cataracts were induced by UV light, cataracts formed in an annular pattern in the cortical equatorial region [15, 20, 39]. In the ex vivo system in which cataracts were induced by H_2_O_2_ or UV light, the white turbidity formed in an annular pattern in the cortical equatorial region, and the area and shape of the white turbidity formation were similar. On the other hand, the changes occurring within the lens when cataracts were induced after 3–4 days of incubation with galactose were significantly different [15]. Lenses with MMS-induced clouding showed a decrease in epithelial cells and an overall decrease in cortical cell density, whereas lenses with galactose-induced clouding formed vacuole-like structures. Thus, it is possible that sugar stress and DNA damage may induce white opacity via different mechanism.

This system also has the potential advantage of excluding off-target effects of UV light that lead to cataract formation, such as photooxidation of lipids and other macromolecules. In fact, by measuring changes in gene expression levels over time in this study, we were able to classify the upregulated genes into four groups according to their expression patterns (Fig 4). Therefore, by using this system, we demonstrated that the expression levels of not only DNA damage response genes but also of other genes that are responsible for the development of cataracts were upregulated even during the preliminary stages of cataractogenesis.

As expected, the expression of genes related to DNA repair increased on the first day after drug treatment, but this should not have any effect on subsequent cataract formation because DNA repair genes are activated immediately after DNA damage occurs [40]. In our system, the expression of genes related to the cell cycle was increased in addition to DNA repair genes, suggesting that factors other than DNA repair mechanism or changes in the balance between DNA repair and the cell cycle could affect cataract induction. In fact, Cdkn1a/p21, one of a group of genes associated with isolated DNA repair (Cdkn1a/P21, Gadd45b, and Mdm2), is a cyclin-dependent kinase (CDK) inhibitor and plays an important role in regulating cell cycle progression [41], in which p21 phosphorylation activates Cdk1 during the G2/M phase [42]. Gadd45b is involved in DNA repair mechanisms such as nucleic acid excision repair, mobilization to DNA damage sites, and regulation of DNA access to repair proteins via interaction with Proliferating Cell Nuclear Antigen (PCNA) [43, 44]. Mdm2 binds to the tumor suppressor p53 and inhibits transcriptional activation of p53 target genes [45]. Cell cycle-related genes include Cdkn1a/p21, Mdm2, and Plk2. Plk2 is activated by the spindle checkpoint during mitosis [46]. Atf3 was increased on Day 1, while a 4-fold increase in Atf3 expression, which is involved in various processes of cellular stress response, was reported in UVA-exposed lens epithelial cells compared to controls [47]. It is possible that Atf3-mediated cellular stress response processes similar to those in the UV-induced model are involved.

Group 2, in which expression peaked Day 2, contained genes involved in oxidative stress and drug efflux, which could be linked to the cell cycle-related genes that were maximally upregulated on Day 1. Genes associated with the oxidative stress response included *Aldh3A1*, *Ephx1*, *Gstp1*, *Mt1a*, *Mt1f*, and *Nqo1*. Aldh3a1 is primarily expressed in the cornea and protects against UV light-induced oxidative damage [48, 49]. Aldh3a1 also has a drug efflux effect that removes reactive aldehydes generated by ROS and lipid peroxidation [50]. Gstp1 is a member of the glutathione transferase family, and binds non-polar compounds to glutathione and transports them out of the cell [51]. Furthermore, *Gstp1* and *Nqo1* are both Nrf2 target genes, and when Nrf2 is activated in response to oxidative stress and *Gstp1* and *Nqo1* are transcribed, the gene products function as ROS detoxification enzymes [52]. Therefore, the relationship between genes related to the oxidative stress response and those related to drug efflux suggests that genes related to oxidative stress response may have been upregulated by the addition of MMS, not only because of their direct response to oxidative stress, but also because of their auxiliary functions in detoxification. Genes associated with drug efflux included *Abcb1b* and *Gstp1*. NQO1 is an antioxidant that reduces quinone to hydroquinone [53, 54], and has been used as a biomarker of oxidative stress [17].

As for the remainder of affected genes, Hmgcs1 and Idi1, which were classified in Group 1, are enzymes in the cholesterol synthesis pathway. Hmgcs1 is a cytoplasmic enzyme located upstream of HMGCR in the mevalonic acid pathway, and produces HMG-CoA from acetyl-CoA and acetoacetyl-CoA [55]. IDI1 produces dimethylallyl diphosphate from isopentenyl diphosphate [56]. SCR rats that are genetically predisposed to cataracts are reported to have mutations in Lss and Fdft1, which encode lanosterol synthase and farnesyl diphosphate farnesyltransferase, respectively, and which, like Hmgcs1 and Idi1, are involved in the cholesterol synthesis pathway [57]. Inhibition of cholesterol synthesis inhibits cell proliferation in LECs [58]. These results suggest that the cholesterol synthesis pathway is potentially involved in the newly established experimental system. It is also possible that *Hmgcs1* and *Idi1*, which were upregulated by MMS, promote the cholesterol synthesis pathway.

The experimental system established in this study, in the which the DNA-damaging agents MMS and Bleomycin induced opacity in explanted rat lenses, is an *ex vivo* experimental system, but is a stable system in which the induction of opacity is both concentration- and time-dependent. Therefore, it is expected to be useful in elucidating the detailed molecular mechanisms of cataracts, and it is expected that more detailed mechanisms will be elucidated in the future through the use of small molecule inhibitors.

## Supporting information

S1 Fig Opacity induced by MMS and Bleomycin. Photographs of all samples used in this study are shown. (A) Opacity induced by incubation of lenses in medium containing MMS. (B) Opacity induced by incubation of lenses in medium containing Bleomycin. In the photograph, “q” on the left denotes the sample used for RT-qPCR, and “M” denotes the sample used for microarray analysis.
(ZIP)

S1 Table List of primer sequences for RT-qPCR. (XLSX)

S2 Table List of genes significantly upregulated in microarray analysis. List of genes with more than 2-fold increase in signal value in MMS-treated (Day 2, Day 4) and Bleomycin-treated (Day 2, Day 6) lenses relative to Control. Probes without corresponding gene names and genes with low signal values (signal value < 5) were excluded from analysis. "◆" indicates genes with a signal value increase > 2-fold relative to Control. The signal values are expressed as log2 changes.
(XLSX)
