# Supplementary figures and images for: Analysis of cataract-regulated genes using chemical DNA damage induction in a rat ex vivo model
Source: PLoS One. 2022 Dec 7;17(12):e0273456. doi: 10.1371/journal.pone.0273456 (PMC9728860; doi:10.1371/journal.pone.0273456)

(A) MMS

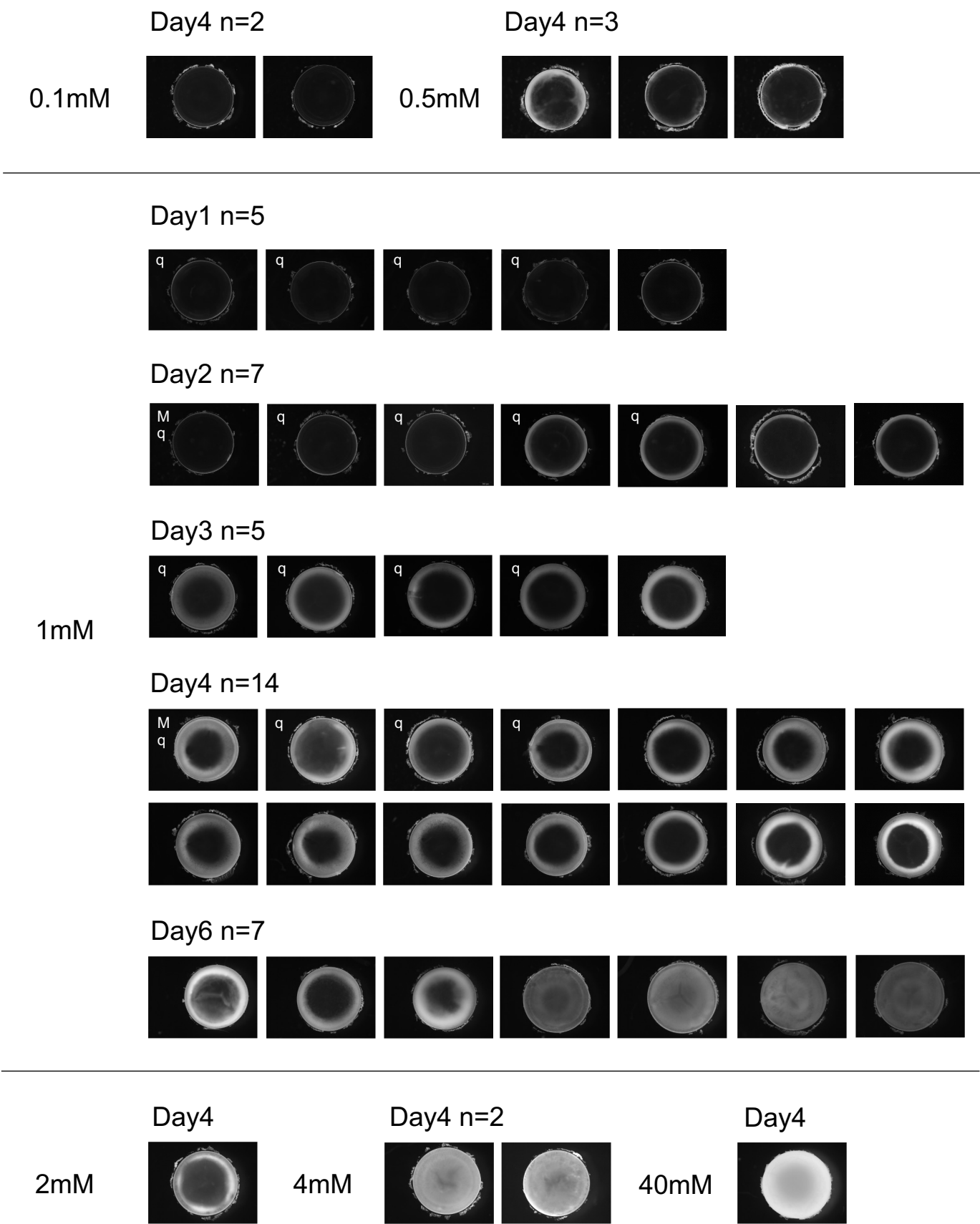

Supplement: S1 Fig — Photographs of all samples used in this study are shown. (A) Opacity induced by incubation of lenses in medium containing MMS. (B) Opacity induced by incubation of lenses in medium containing Bleomycin. In the photograph, “q” on the left denotes the sample used for RT-qPCR, and “M” denotes the sample used for microarray analysis. (ZIP) [file pone.0273456.s001.zip › S1A_Fig.pdf]

(B) Bleomycin

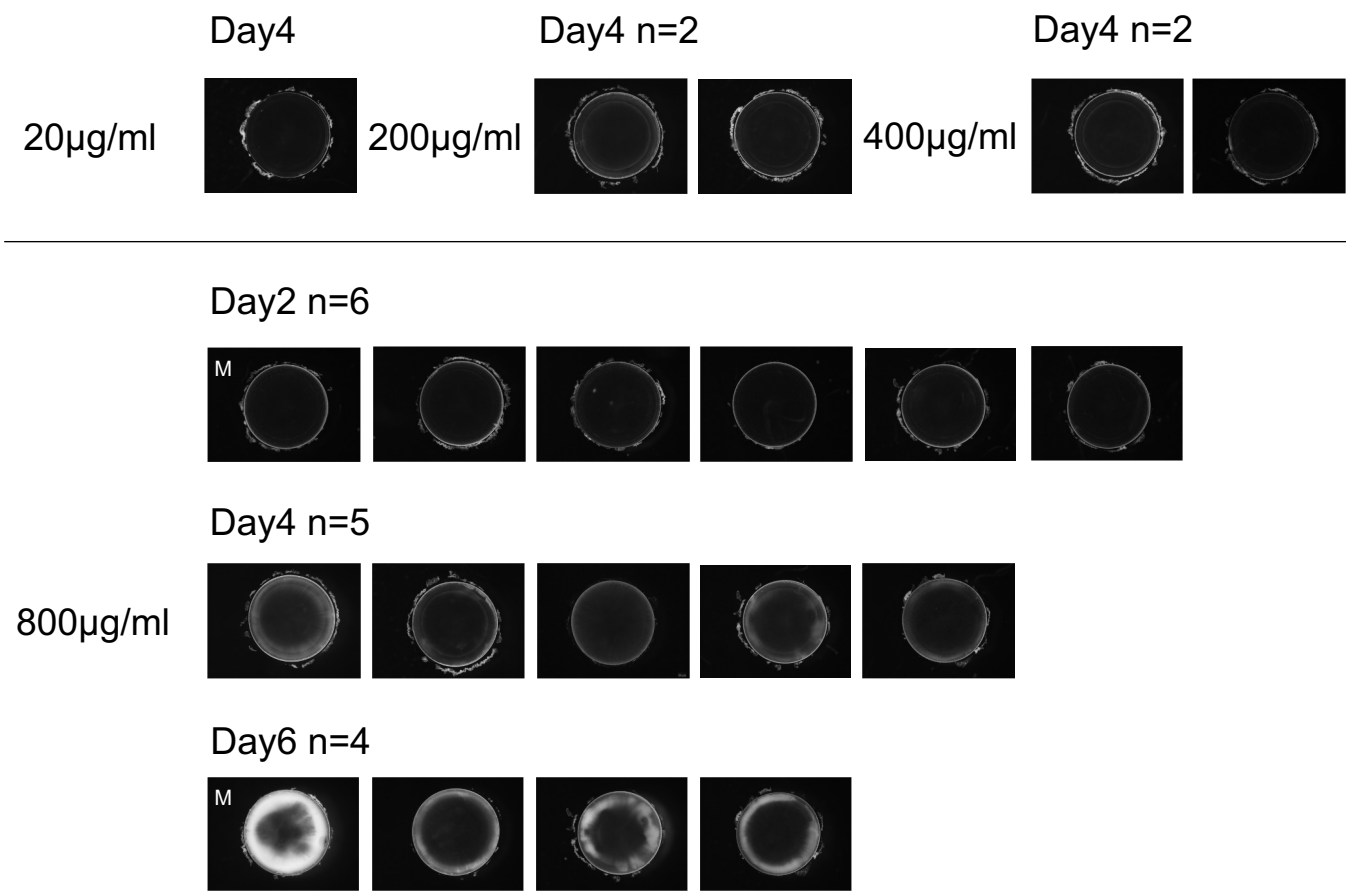

Supplement: S1 Fig — Photographs of all samples used in this study are shown. (A) Opacity induced by incubation of lenses in medium containing MMS. (B) Opacity induced by incubation of lenses in medium containing Bleomycin. In the photograph, “q” on the left denotes the sample used for RT-qPCR, and “M” denotes the sample used for microarray analysis. (ZIP) [file pone.0273456.s001.zip › S1B_Fig.pdf]
